# Targeted Analysis of Placental Steroid Hormones in Relation to Maternal Tobacco Smoke Exposure: Early Markers Relevant to DOHaD (Developmental Origins of Health and Disease)

**DOI:** 10.3390/ijms262110548

**Published:** 2025-10-30

**Authors:** Alicja Kotłowska, Sebastian Fitzek, Rafał Stettner, Sylwia Narkowicz, Bogumiła Kiełbratowska, Piotr Szefer

**Affiliations:** 1 Department of Clinical and Experimental Endocrinology, Faculty of Health Sciences, Medical University of Gdańsk, Dębinki 7, 80-210 Gdańsk, Poland; 2 Department of Food Sciences, Faculty of Pharmacy, Medical University of Gdańsk, Al. Gen. J. Hallera 107, 80-416 Gdańsk, Poland; piotr.szefer@gumed.edu.pl; 3 Faculty of Communication and Public Relations, National University of Political Studies and Public Administration, 012104 Bucharest, Romania; sebastian.fitzek@comunicare.ro; 4 Institute for Quality of Life Research, Romanian Academy, 050711 Bucharest, Romania; 5 Clinical Department of Operative Gynecology and Gynecologic Oncology, University Hospital 4 Dr. K. Jaczewskiego 8, 20-090 Lublin, Poland; rafalstettner@gmail.com; 6 Department of Analytical Chemistry, Chemical Faculty, Gdańsk University of Technology, 11/12 Narutowicza St., 80-233 Gdańsk, Poland; sylwianarkowicz@10g.pl; 7 Department of Obstetrics, Faculty of Medical Sciences, Medical University of Gdańsk, Dębinki 7, 80-210 Gdańsk, Poland; bogumila.kielbratowska@gumed.edu.pl

**Keywords:** placenta, steroid hormones, tobacco smoke, passive smoking, DOHaD, HPLC–Corona CAD, developmental origins, endocrine disruption

## Abstract

Maternal tobacco smoke exposure is associated with impaired fetal growth and long-term disease risk (DOHaD, Developmental Origins of Health and Disease). Whether placental steroid hormones are independently altered remains a matter of debate. We quantified six placental steroids (estradiol, estriol, estrone, progesterone, testosterone, and pregnanediol) using HPLC–Corona CAD in 70 deliveries (C = 30; PS = 20; AS = 20). Distributional differences were assessed with Kruskal–Wallis and pairwise Mann–Whitney tests with Benjamini–Hochberg (BH) control. Adjusted associations used log-linear OLS with HC3 robust SE: Model A (gestational age, maternal BMI, newborn sex) and Model B (Model A + birth weight), reported as percent change vs. controls, computed as (exp(β) − 1) × 100 with 95% CI. Secondary analyses tested (i) multiclass logistic classification of C/PS/AS from the steroid panel (5-fold stratified CV) and (ii) prediction of birth weight (OLS and 2-component PLS). All six steroids differed by group (BH-adjusted p ranging from 9.18 × 10^−12^ to 6.66 × 10^−8^). In Model A, AS vs. C showed lower estrogens/progestins (estradiol, −46.2%; estriol, −24.7%; estrone, −25.9%; progesterone, −28.2%; pregnanediol, −31.4%) and higher testosterone (+40.8%); these effects persisted in Model B after adjusting for birth weight. The panel classified C/PS/AS with 0.900 cross-validated accuracy (weighted OvR AUC 0.994). Hormones poorly predicted birth weight (PLS CV R^2^ = −0.777). Maternal active and passive smoking is associated with a coherent and independent disruption of placental steroidogenesis. A targeted placental steroid panel offers biologically meaningful early markers relevant to DOHaD.

## 1. Introduction

It has been recently shown that the status of the fetus during pregnancy might be directly related to the development of several adult diseases [1]. Exposure to various extrinsic factors during the critical intrauterine period, when the fetus is more susceptible, may result in negative health outcomes in childhood and adulthood, including malnutrition or overnutrition, altered growth, and mental or metabolic diseases. These observations were first described by Barker et al. [2], who proposed the hypothesis of fetal origins of adult diseases, which later served as a foundation for the theory of developmental origins of health and disease (DOHaD) [2]. The so-called fetal programming has been associated with the response of the fetus to the intrauterine environment or the factors that affect it. Fetal programming may induce both structural and functional changes in cells, tissues, and organs during critical developmental periods and result in negative long-term consequences for health and the development of diseases [3]. The changes described above might be associated with the development of hypertension, cardiovascular diseases, insulin resistance, or obesity, just to name a few. Among the most important factors associated with fetal programming are exposure to infectious agents, mutagens, and toxins, availability of nutrients, and endocrine disorders in mothers [4]. To add, long-term consequences of prenatal tobacco exposure in offspring include: development of asthma, poor lung function, neurodevelopmental and behavioral disorders such as ADHD, poorer cognitive/academic outcomes, increased obesity, and cardiometabolic risk. These effects are supported by epidemiologic studies, meta-analyses, and mechanistic work showing persistent epigenetic changes [5,6].

Cigarette smoke and its components may be responsible for alterations in the processes associated with fetal programming. At present, over 1.3 billion people worldwide smoke cigarettes [7], and even more individuals are exposed to environmental tobacco smoke (ETS) exhaled by smokers. It is estimated that approximately 5 million people die each year as a result of tobacco smoke exposure and smoking-related illnesses [8], as cigarette smoke (CS) contains over 4800 compounds, including at least 200 toxic and genotoxic agents, more than 69 carcinogens [9], and a variety of endocrine-disrupting chemicals (EDCs) and metalloids. Knowledge concerning the negative effects of smoking on the fetus and the newborn child is increasing; nevertheless, this habit remains an important public health issue worldwide, as approximately 15–25% of women smoke during pregnancy [10,11]. CS exposure during gestation has been associated with several pre- and postnatal complications and adverse maternal, fetal, and neonatal outcomes [12,13]. Studies have suggested that maternal smoking is responsible for spontaneous miscarriages, placental abruption, placenta previa, fetal death [14,15], preterm delivery, low birth weight, impaired postnatal growth, sudden infant death syndrome (SIDS), and many behavioral abnormalities in infants [16]. In addition, maternal exposure to cigarette smoke during pregnancy may alter the synthesis and levels of various fetal, maternal, and placental hormones. The placenta is of particular interest in studies on prenatal cigarette smoke exposure [17,18]. Its major function is to transport molecules between the mother and fetus, allowing nutrient uptake, eliminating waste products, and supporting gas exchange for the fetus. Due to these properties of the organ, the compounds present in cigarette smoke can freely pass through the placental barrier and reach the fetus [19]. The placenta is also responsible for the production of many hormones that affect the ovaries, uterus, and fetus. Steroid hormones are of utmost importance as they play a critical role in the progression of pregnancy, fetal survival, and development, as well as in their well-being [20,21]. Nevertheless, knowledge about the effect of maternal cigarette smoking on the steroid hormones produced by the placenta remains limited. In addition, most studies concentrate only on the influence of CS on two compounds, progesterone and estradiol [22], and do not measure the impact of tobacco smoke on the placental hormonal profile.

Recent advances in hyphenated liquid chromatography techniques have enabled the robust targeted quantification of endogenous molecules, supporting biomarker studies within the DOHaD framework [19,20]. Here, we focused on a targeted design: precise quantification of a defined six-steroid placental panel measured by HPLC–Corona CAD, followed by prespecified statistical analyses. Considering all of the above, this study examined the relationship between maternal tobacco smoke exposure during gestation and alterations in placental steroid hormone profiles, recognizing these hormonal disruptions as pivotal early markers within the DOHaD paradigm. In this study, we analyzed a targeted placental steroid panel—estradiol, estriol, estrone, progesterone, pregnanediol, and testosterone—to evaluate alterations associated with maternal tobacco smoke exposure.

## 2. Results

### 2.1. Study Groups

**Figure 1:**
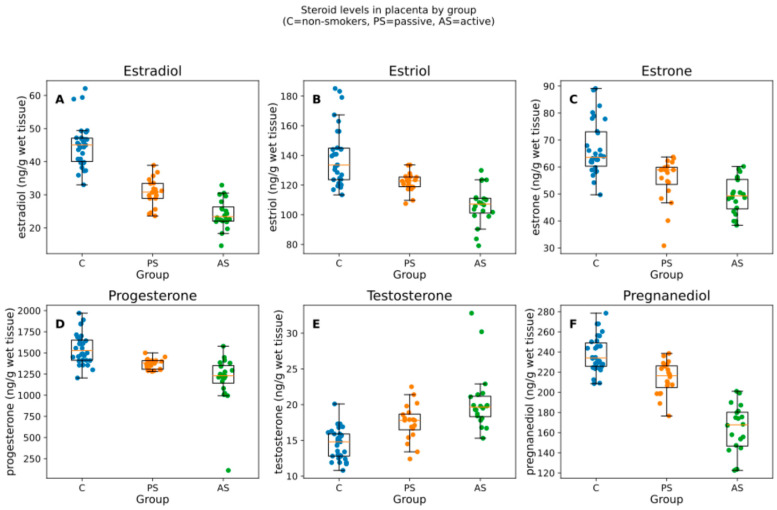
Box-and-jitter plots of the six placental steroid hormones by group. Boxes show the IQR with median; whiskers indicate the non-outlier range; points are individual samples. Units: ng/g of wet tissue. Panels (**A**–**F**) correspond to estradiol, estriol, estrone, progesterone, testosterone, pregnanediol. C = non-smokers, PS = passive smokers, AS = active smokers.

The study population comprised mainly women with higher education levels, who regularly ate dairy and vegetable products and rarely consumed alcohol 1) with a BMI above 20 and below 25. The mean weight of the babies was between 3215 and 3764 g (Table 1). We analyzed 70 placentas (C = 30, PS = 20, AS = 20). All six steroids differed by group (Kruskal–Wallis; BH-adjusted p from 9.18 × 10^−12^ to 6.66 × 10^−8^). The median and interquartile range [IQR] by group are summarized in Table 2 and visualized in Figure 1.

### 2.2. Analytical Method

Method validation included linearity, LOD, LOQ, precision, and accuracy using QC (quality control) samples employing steroid-free placental extracts obtained by stripping the extracts of the compounds using charcoal. Calibration curves were constructed for the model compounds by applying quality control samples fortified with reference standards at different concentrations. The method was linear in the range of 30–500 ng/mL (R^2^ = 0.997–0.998). The LOD values for the tested compounds were between 18 and 20 ng/mL, whereas the LOQ values were found in the range between 30 and 33 ng/mL. Within-day precision, expressed as % CV, ranged from 13.5% to 4% and accuracy was 87–108% (Table A2). The between-day precision ranged from 4% to 14% and accuracy was found to be between 83–105%. The extraction recoveries ranged from 78–94% for all steroids (Table A2). The method was verified for selectivity by analyzing blank samples of steroid-free placenta extract (matrix) for the presence of any interfering compounds. The method was found to be selective, as no interfering compounds were detected during the analyses.

### 2.3. Primary Statistics Results

**Figure A1:**
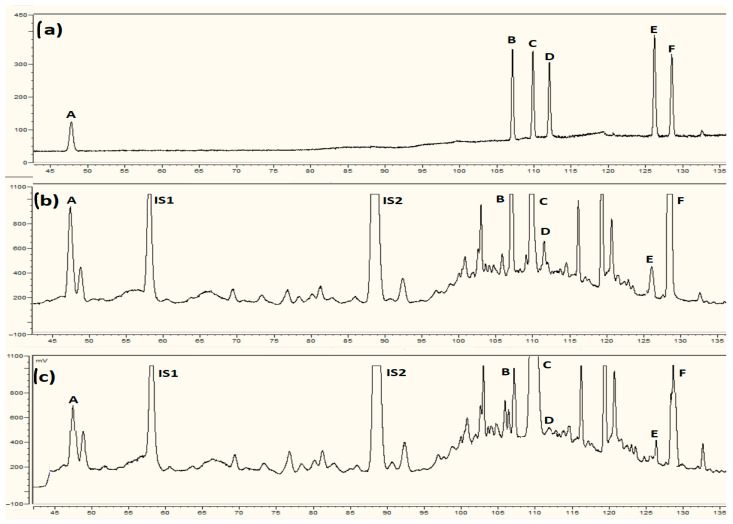
Separation of placental steroid hormones by HPLC-Corona CAD: (**a**) standard mixture, (**b**) sample from non-smoking control, (**c**) sample from smoking mother. The compounds detected were A—estriol, B—17β-estradiol, C—testosterone, D—estrone, E—pregnanediol, F—progesterone, IS1—internal standard dexamethasone, and IS2—internal standard nandrolone.

The validated quantitative method was applied to measure the levels of steroid hormones in the placental samples. Six steroids were detected in each sample (Figure A1). The concentrations of 17β-estradiol, estriol, estrone, progesterone, pregnanediol, and testosterone were measured in the placenta samples of non-smoking controls, passive smokers, and active smokers. The results of the Kruskal–Wallis test for non-parametric group comparisons with BH correction are shown in Table 2. Boxplots and visual representations of the Kruskal–Wallis test results are presented in Figure 1. Pairwise Mann–Whitney tests (BH within hormone) confirmed graded differences; full U and adjusted *p*-values are shown in Table 3. Complete per-comparison statistics and full regression outputs are provided in Supplementary Table S2.

The analysis of steroid levels in placentas of non-smoking controls, passive smokers, and active smokers applying Kruskal–Wallis test indicated that the overall production of 17β-estradiol, estriol, estrone, progesterone, and pregnanediol (metabolite of progesterone) was statistically significantly decreased in the active smoker groups when compared with non-smokers (KWp-BH < 0.0001) and the concentrations were equal to 23.40 ng/g wet tissue, 107.20 ng/g wet tissue, 49.30 ng/g wet tissue, 1229.55 ng/g wet tissue, and 167.70 ng/g wet tissue in the AS group, respectively (Table 2, Figure 1). The decrease was lower in the passive smoker group and higher in the active smoker group. The levels of testosterone were significantly higher (*p* < 0.0001) in the samples of smokers than in those of non-smokers and reached peak values for active smokers equal to 19.65 ng/g wet tissue (Table 2).

### 2.4. Adjusted Models

**Figure 2:**
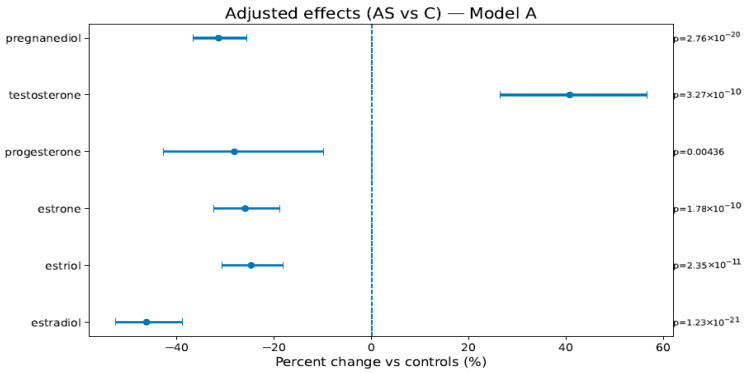
Adjusted percent change (AS vs. C) in hormone levels (Model A: weeks, BMI, and newborn sex). Points are estimates; bars are 95% CIs; the dashed line is at 0%. The right margin shows the HC3-robust *p*-values. The BH-FDR was applied as described in Section 4.4.1.

**Figure 3:**
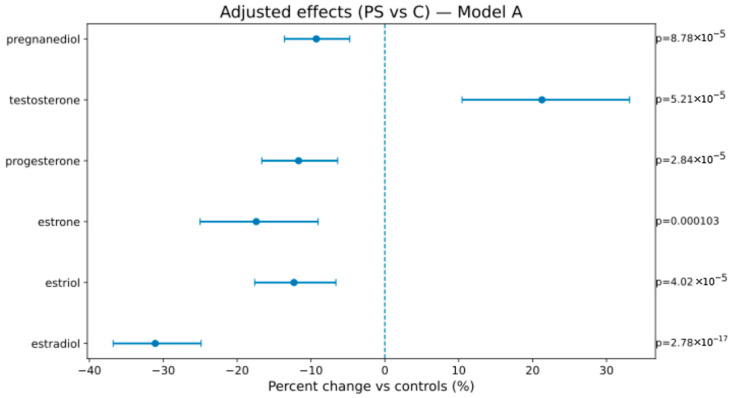
Adjusted percent change (PS vs. C) in hormone levels (Model A). The display conventions are the same as those in Figure 2.

In Model A (weeks, BMI, sex), AS vs. C showed lower estrogens/progestins—estradiol −46.2% [−52.7; −38.9], estriol −24.7% [−30.7; −18.2], estrone −25.9% [−32.5; −18.8], progesterone −28.2% [−42.8; −9.8], pregnanediol −31.4% [−36.7; −25.7]—and higher testosterone +40.8% [+26.5; +56.6]; PS vs. C showed attenuated but direction-consistent changes (see Figure 1, Figure 2 and Figure 3 and Table 4). In Model B (adding birth weight), all directions and significances persisted (Supplementary Figures S1 and S2; Supplementary Table S1), supporting independence from birth weight.

### 2.5. Multi-Class Classification

Using ln-transformed, z-standardized steroid concentrations, a multinomial logistic (one-vs-rest) classifier achieved 0.900 mean cross-validated accuracy, and 0.994 weighted OvR AUC (5-fold stratified CV; seed = 42) (Table 5). The mean CV confusion matrix (rows = true; columns = predicted) was C → (29, 1, 0), PS → (3, 15, 2), and AS → (0, 1, 19). Class-wise metrics were C precision/recall/F1 = 0.91/0.97/0.94; PS = 0.88/0.75/0.81; AS = 0.90/0.95/0.93. Misclassifications were concentrated in the PS, consistent with biological adjacency. This analysis is exploratory, given the sample size.

Hormones showed limited predictive value for birth weight: OLS adjusted R^2^ = 0.291 (weeks significant; hormones not), and PLS (2 components) 5-fold CV R^2^ = −0.777, indicating poor out-of-sample prediction (Table 6).

## 3. Discussion

The six-steroid pattern of decreased estrogens/progestins and increased testosterone is compatible with altered placental steroidogenesis under tobacco exposure, potentially reflecting shifts in aromatase activity and luteo-placental function. Importantly, Model B demonstrated that these differences were not explained by birth weight, supporting their interpretation as markers of pathway disruption rather than mere proxies of fetal growth.

Steroid hormones play a vital role in the regulation of several processes during pregnancy and the development of the fetus, and disruption in their production due to fetal programming might have serious consequences in terms of fetal programming and future development of diseases in adult life. Estrogens exert a dual effect by either sustaining pregnancy during its earlier stages or inducing labor later. Other effects of estrogens include stimulation of angiogenesis [21] and increased progesterone levels [22]. In more advanced stages of pregnancy, these compounds aid in preparing the uterus and other tissues for parturition. Moreover, estrogens promote fetal maturation and the development of the lungs, liver, and other organs and tissues [23,24]. Other steroid hormones crucial for maintaining pregnancy include progesterone and its derivatives, such as pregnanediol. They are responsible for inhibiting the mother’s immune system and blocking the process of rejection of the fetus. In most mammals, a drop in circulating progesterone levels induces preterm labor [25]. Finally, testosterone induces fetal genital development in male fetuses. However, studies have revealed that increasing levels of circulating maternal testosterone are correlated with various disorders in adulthood [26,27].

This study aimed to determine the effect of maternal smoking on fetal programming based on steroid profiling in human placentas using a hyphenated technique. The concentrations of six steroid hormones (17β-estradiol, estriol, estrone, progesterone, testosterone, and pregnanediol) in samples from non-smokers differed from those in samples from active and passive smokers. The concentrations of 17β-estradiol, estriol, estrone, progesterone, testosterone, and pregnanediol were highest in the placentas of non-smoking mothers and gradually decreased in the placentas of passive smokers. The lowest concentrations of these compounds were found in the samples from active smokers. Similar findings for progesterone levels in smoking mothers have been reported by other researchers [20]. Cigarette smoking by expectant mothers alters essential aspects of placental function, such as progesterone production [20], estrogen metabolism [28], and placental steroid disruption, and this process is affected by metal exposure. This observation can be explained by the fact that cigarette smoke is a source of Cd ions, which accumulate in the human placenta and inhibit progesterone release; thus, the increase in placental Cd is correlated with a decrease in placental progesterone concentrations. The negative effects of active and passive smoking on estrogen production have also been observed in premenopausal women [29]. Exposure to cigarette smoke has a dual effect on the synthesis of these compounds. First, alkaloid derivatives from tobacco smoke inhibit aromatase cytochrome P-450 activity and suppress the conversion of androgens to estrogens [30,31]. Moreover, CS influences the process of 2-hydroxylation of estradiol metabolites, resulting in the increased production of 2-hydroxy estrogens. These compounds are irreversible metabolites, possess very low estrogenic activity, and are rapidly eliminated from the organism. Lower levels of estriol and other estrogens in the placental samples from smokers can also be explained by the suppression of the activity of placental aromatase and possibly 16α-hydroxylase in the fetal liver. Current studies have also indicated that smoking may significantly increase androgen levels in females [32,33]. Our study revealed that testosterone levels increased in the placentas of passive smokers and peaked in the samples from active smokers. The increase in androgen concentration may be explained by the inhibition of placental aromatase activity [34]. This increase in testosterone not only suggests a direct impact on the development of fetal gonads but can also be associated with long-term health concerns, as elevated testosterone levels in adult women are related to cardiovascular and metabolic issues [35]. In women of reproductive age, heavy metals in CS, categorized as endocrine-disrupting chemicals, can directly interfere with steroid hormone synthesis pathways, inducing enzymes such as aromatase or modulating 3β-hydroxysteroid dehydrogenase (3β-HSD) activity, further skewing the hormonal milieu [36]. This interference can lead to premature reproductive aging and an increased risk of early menopause in female offspring due to compromised ovarian function and altered steroid hormone profiles. To add, it is worth emphasizing that the harmful compounds present in tobacco smoke exert other adverse health effects. Carbon monoxide (CO) binds to hemoglobin in the blood and forms carboxyhemoglobin, which is responsible for the reduction of oxygen delivery and leads to tissue hypoxia. Nicotine stimulates the release of stress hormones (adrenaline, noradrenaline), which cause vasoconstriction, faster heart rate, and elevated blood pressure [37]. Moreover, CS contains various oxidants and free radicals that promote oxidative stress, damaging vascular endothelium, impairing nitric oxide–mediated vasodilation, and inducing DNA mutations [38]. These factors influence placental perfusion and hormone biosynthesis. The results of our study indicate that maternal exposure to cigarette smoke alters the steroid hormone profile in the placenta. Additionally, these compounds may serve as biomarkers associated with the Developmental Origins of Health and Disease (DOHaD) framework. The changes in steroid concentrations highlight the pathway by which exposure to tobacco smoke may program future disease risk in offspring. Logistic regression analysis revealed that all six studied steroids are markers associated with DOHaD mechanisms, rather than simply reflecting recent exposure to tobacco smoke (in this case, the measurement of cotinine is sufficient). The multi-class classifier is presented as proof-of-concept for biological discrimination across C/PS/AS based on the steroid panel, and not as a substitute for exposure biomarkers (e.g., cotinine). The panel captures changes at the maternal–fetal interface (DOHaD relevance). External validation in larger, multicenter cohorts is warranted. Additionally, hormonal changes exert effects beyond the outcome of reduced birth weight and may indicate deeper mechanisms of endocrine disruption. This observation was confirmed by the analysis of a transparent multinomial logistic model with a 5-fold stratified CV. Birth weight was excluded from classifiers (redundancy avoidance) and retained only as a covariate in OLS to test independence. Finally, when predicting birth weight using PLS regression, a negative cross-validated value of R^2^ was obtained, reinforcing the statement that steroid disruption is not merely an indirect marker of fetal growth restriction. Instead, hormonal changes reflect distinct biological processes that cannot be fully captured by birth weight alone, outlining the multifactorial impact of maternal tobacco exposure.

The main limitation of the study is the limited sample size and lack of data concerning the influence of maternal diet and the possibility of residual confounding from unmeasured factors such as maternal diet or co-exposures. In order to serve as a meaningful clinical predictive tool, the selected steroid panel acting as early biomarkers relevant to the DOHaD, the findings of the study should be validated using a larger sample size.

## 4. Materials and Methods

### 4.1. Ethics Statement

All participants provided informed consent, and the study was approved by the Local Ethical Committee of the Medical University of Gdańsk, Poland (reference number NKEBN/80/2010, 10 July 2010).

### 4.2. Design and Groups

The study group was recruited from patients at the Department of Obstetrics, Faculty of Medical Sciences, Medical University of Gdańsk, Poland. The subjects included 70 Polish pregnant women divided into three groups: non-smokers (C, N = 30), passive smokers (PS, N = 20), and active smokers (AS, N = 20). The women included in the study were not treated with any steroid-like medications and did not have any endocrine disorders. The only medication allowed was painkillers, and subjects using medication disrupting steroid hormone pathways were excluded. The participating women completed a questionnaire on lifestyle factors, including smoking (frequency, active or passive smoking, and diet). The smoking status of mothers was self-reported and also verified by the measurement of thiocyanate ion levels in placenta samples using ion chromatography, with the levels of the compound being higher in smokers than in non-smokers (data reported in “Determination of thiocyanate as a biomarker of tobacco smoke constituents in selected biological materials of human origin” by Narkowicz et al. [39]). Groups were defined as non-smokers (C), passive smokers (PS), and active smokers (AS); the final sample sizes were C = 30, PS = 20, and AS = 20 (total n = 70).

All participants provided written informed consent prior to participation in the study. The women were aged between 21 and 40 years, and their BMI before pregnancy was between 19–25. The babies were delivered healthy after full-term pregnancies (37–41 weeks) via vaginal birth.

### 4.3. Sample Handling, Analytes, and Assay

**Figure 4:**
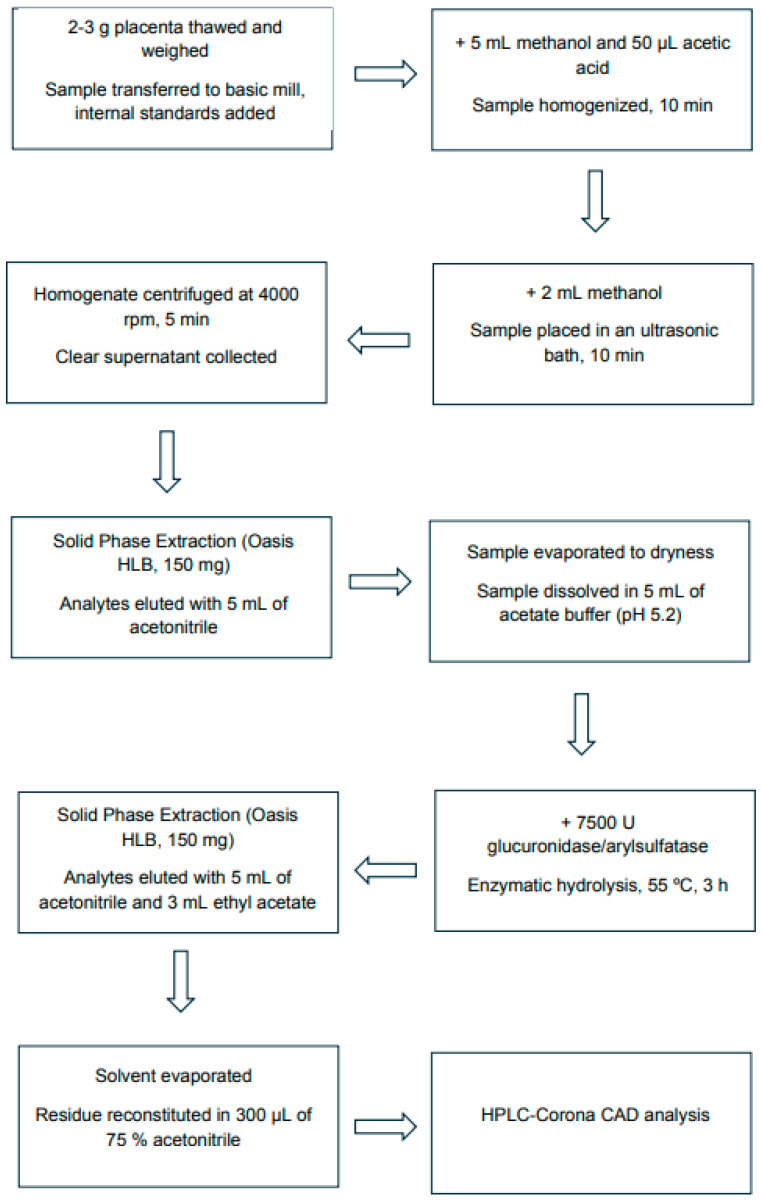
Flow diagram of the analytical procedure for sampling and steroid hormone extraction from the human placenta.

Placenta samples were collected from mothers at parturition. Tissue was collected <10 min post-delivery, rinsed with isotonic saline, and central cotyledon samples were snap-frozen and stored at −70 °C until extraction. Cross-sectional analysis of placental tissue at delivery: controls (C), passive smokers (PS), and active smokers (AS). Steroid hormones were extracted from placental samples according to a modified procedure [40], as presented in Figure 4.

All experiments were carried out using an HPLC Ultimate 3000 system (Dionex, Germering, Germany), consisting of a quaternary pump, an autosampler, and a column heater. The system was coupled to a Corona CAD detector (ESA, Chelmsford, MA, USA). Data processing was performed using Chromeleon 6.8 software (Dionex, Germering, Germany). The nitrogen gas flow rate was regulated automatically and monitored by the CAD device and was supplied by a nitrogen generator Sirocco-5 (Schmidlin-DBS, Geneva, Switzerland), regulated at 35 psi. The response range was set to 20 pA full scale. A medium filter was applied. Chromatographic analysis was performed at 25 °C. Samples were separated on a Thermo Hypersil (Thermo Fisher Scientific, Waltham, MA, USA) C18 column packed with 5 μm shell particles (150 × 4.6 mm). The mobile phase consisted of acetonitrile (solvent A) and 0.1% trifluoroacetic acid (solvent B), and elution was performed by programming the concentration of the mobile phase. The flow rate was 1 mL/min, and the injection volume was 10 μL. A 140 min gradient program was used (Table A1). Each sample was analyzed in triplicate, and the peaks corresponding to steroid hormones in the chromatograms were identified by comparing the retention times and UV spectra with those of the standards. Validation was performed using a placenta matrix passed through an Oasis HLB cartridge spiked with a standard concentration of including 17β-estradiol, estriol, estrone, progesterone, pregnanediol testosterone, dexamethasone (IS),) and nandrolone (IS2). Calibration curves of the standards were obtained in the concentration range of 30–5000 ng/mL for each compound.

Six steroid hormones present in the placenta samples were quantified by HPLC–Corona CAD, and the results were calculated as ng/g wet tissue, normalized to tissue wet mass.

### 4.4. Statistics

Analyses used Python 3.11.13 with numpy 2.3.3, pandas 2.3.2, scipy 1.16.2, statsmodels 0.14.5, and scikit–learn 1.7.2 (random seed = 42). All codes and exact environment specifications are archived with the dataset (see Data Availability).

#### 4.4.1. Primary Statistics

Group differences for each hormone were assessed using Kruskal–Wallis tests. Pairwise comparisons were performed using two-sided Mann–Whitney tests. To control for multiplicity, the Benjamini–Hochberg false discovery rate (BH–FDR) was applied (i) across the six Kruskal–Wallis tests and (ii) within each hormone across its three pairwise comparisons.

#### 4.4.2. Adjusted Models

We fitted OLS models on ln-transformed hormone levels with heteroskedasticity-consistent HC3 standard errors. Complete regression outputs are provided in Supplementary Table S2. Model A was adjusted for gestational weeks at delivery, pre-pregnancy maternal BMI, and newborn sex; Model B was additionally adjusted for birth weight (kg). Effects are reported as percent change relative to controls, computed as (exp(β) − 1) × 100, with 95% confidence intervals obtained by transforming β ± 1.96·SE back to the original scale. Complete regression outputs are provided in Supplementary Table S2.

#### 4.4.3. Secondary Analyses

For multi-class discrimination of C/PS/AS, we trained a multinomial logistic regression (One-vs-Rest) on ln-transformed, z-standardized hormone features (six variables). Performance used 5-fold stratified cross-validation (seed = 42), reporting accuracy, weighted OvR ROC AUC, the cross-validated confusion matrix, and class-wise precision/recall/F1. Birth weight was not used as a predictor to avoid redundancy or leakage. For the prediction of birth weight, we fitted OLS (weeks, BMI, sex, ln-hormones) and PLS regression (two components) with 5-fold CV. Complete case analysis; no imputation.

## 5. Conclusions

Maternal active and passive smoking is associated with a robust, biologically plausible pattern of decreased estrogen/progestin and increased testosterone levels in the placenta, independent of birth weight. A targeted steroid panel offers interpretable early markers relevant to the DOHaD. However, additional analyses and a larger sample size are required in order to be validated as a meaningful predictive tool in clinical practice.

## Figures and Tables

**Table 1:** Characteristics of the study group.

Characteristic	Non-Smoking	Passive Smokers	Active Smokers
	(N = 30)	(N = 20)	(N = 20)
**Age (range)**	21–35	21–35	21–35
**BMI (median, interquartile range)**	20.89	21.57	23.14
	(20.02–23.70)	(20.20–22.57)	(21.63–24.03)
**Smoking (number of cigarettes/24 h)**			
1–5	0	0	3
6–11	0	0	13
11–15	0	0	4
**Passive exposure (at least twice a week)**	0	20	0
**Gender of the baby**			
Female	14	9	9
Male	16	11	11
**Apgar score (median, SD)**	8.87 ± 0.78	8.9 ± 1.02	8.75 ± 0.79
**Weeks of pregnancy (median, interquartile range)**	41.00	39.00	39.00
	(39.00–41.00)	(38.00–40.00)	(38.00–41.00)
**Weight of the baby (g)**	3745	3305	3125
**(median, interquartile range**)	(3540–3930)	(3080–3400)	(3090.00–3460)
**Education level**			
Elementary	0	0	0
Secondary	2	1	1
Higher education	28	19	19
**Consumption of dairy products**			
Everyday	29	20	20
3 times a week	1	0	0
**Consumption of vegetables**			
Everyday	30	20	19
3 times a week	0	0	1
**Consumption of alcohol**			
Once a month	2	2	1

**Table 2:** Medians [IQR] and Kruskal–Wallis test (H, p, BH–p) for each hormone (ng/g wet tissue). Controls (C) n = 30; passive smokers (PS) n = 20; active smokers (AS) n = 20.

Hormone [ng/g Wet Tissue]	C (Median [IQR])	PS (Median [IQR])	AS (Median [IQR])	KW_H	KW_p	KW_p_BH
Estradiol	45.05 [40.05; 47.10]	30.80 [28.90; 33.42]	23.40 [22.05; 26.32]	54.41	1.53 × 10^−12^	9.18 × 10^−12^
Estriol	133.50 [123.65; 144.88]	122.80 [118.90; 125.42]	107.20 [101.12; 111.08]	34.65	2.99 × 10^−8^	3.59 × 10^−8^
Estrone	63.55 [60.30; 73.05]	58.90 [53.52; 59.90]	49.30 [44.47; 55.38]	35.20	2.27 × 10^−8^	3.59 × 10^−8^
Progesterone	1528.10 [1413.50; 1651.47]	1383.40 [1306.50; 1408.75]	1229.55 [1142.60; 1350.30]	33.05	6.66 × 10^−8^	6.66 × 10^−8^
Testosterone	14.80 [12.80; 15.90]	17.80 [16.48; 18.68]	19.65 [18.32; 21.18]	34.71	2.90 × 10^−8^	3.59 × 10^−8^
Pregnanediol	234.10 [225.80; 249.03]	216.40 [204.68; 226.23]	167.70 [146.65; 180.20]	48.89	2.42 × 10^−11^	7.25 × 10^−11^

**Table 3:** Pairwise Mann–Whitney tests (U, raw p, BH–p). C = non-smokers; PS = passive smokers; AS = active smokers.

Hormone	Pair	U	p_raw	p_adj_BH
Estradiol	C vs. AS	600.00	3.00 × 10^−9^	9.00 × 10^−9^
Estradiol	C vs. PS	589.00	1.10 × 10^−8^	1.65 × 10^−8^
Estradiol	PS vs. AS	346.00	8.27 × 10^−5^	8.27 × 10^−5^
Estriol	C vs. AS	566.50	1.38 × 10^−7^	4.14 × 10^−7^
Estriol	C vs. PS	465.00	1.12 × 10^−3^	1.12 × 10^−3^
Estriol	PS vs. AS	336.00	2.46 × 10^−4^	3.69 × 10^−4^
Estrone	C vs. AS	571.00	8.45 × 10^−8^	2.54 × 10^−7^
Estrone	C vs. PS	495.00	1.16 × 10^−4^	1.74 × 10^−4^
Estrone	PS vs. AS	300.00	7.09 × 10^−3^	7.09 × 10^−3^
Pregnanediol	C vs. AS	600.00	3.00 × 10^−9^	9.00 × 10^−9^
Pregnanediol	C vs. PS	504.00	5.56 × 10^−5^	5.56 × 10^−5^
Pregnanediol	PS vs. AS	385.00	6.01 × 10^−7^	9.01 × 10^−7^
Progesterone	C vs. AS	548.00	9.51 × 10^−7^	2.85 × 10^−6^
Progesterone	C vs. PS	500.50	7.47 × 10^−5^	1.12 × 10^−4^
Progesterone	PS vs. AS	316.00	1.78 × 10^−3^	1.78 × 10^−3^
Testosterone	C vs. AS	32.50	1.23 × 10^−7^	3.69 × 10^−7^
Testosterone	C vs. PS	103.50	1.03 × 10^−4^	1.55 × 10^−4^
Testosterone	PS vs. AS	101.00	7.66 × 10^−3^	7.66 × 10^−3^

Note: Under BH–FDR, adjusted p is ≥the corresponding raw p by construction; adjustment is performed within each hormone across its three pairwise comparisons (see Section 4.4.1).

**Table 4:** Adjusted effects (Model A): % change [95% CI], robust *p*-value. C = non-smokers, AS = active smokers, PS = passive smokers. Model A (weeks + BMI + sex).

Hormone	AS vs. C	PS vs. C
Estradiol	−46.2% [−52.7; −38.9] (*p* = 1.23 × 10^−21^)	−31.1% [−36.8; −24.9] (*p* = 2.78 × 10^−17^)
Estriol	−24.7% [−30.7; −18.2] (*p* = 2.35 × 10^−11^)	−12.3% [−17.6; −6.6] (*p* = 4.02 × 10^−5^)
Estrone	−25.9% [−32.5; −18.8] (*p* = 1.78 × 10^−10^)	−17.4% [−25.0; −9.0] (*p* = 1.03 × 10^−4^)
Progesterone	−28.2% [−42.8; −9.8] (*p* = 0.00436)	−11.7% [−16.7; −6.4] (*p* = 2.84 × 10^−5^)
Testosterone	+40.8% [+26.5; +56.6] (*p* = 3.27 × 10^−10^)	+21.3% [+10.4; +33.1] (*p* = 5.21 × 10^−5^)
Pregnanediol	−31.4% [−36.7; −25.7] (*p* = 2.76 × 10^−20^)	−9.3% [−13.6; −4.8] (*p* = 8.78 × 10^−5^)

**Table 5:** Multiclass classification (overall metrics, confusion matrix, class-wise metrics). (A). Overall performance. (B). Mean CV confusion matrix, aggregated across 5 folds (rows = true class; columns = predicted class). (C). Class-wise CV metrics (approximate).

**(A)**			
**Metric**		**Value**	
Accuracy		0.900	
weighted AUC (OvR)		0.994	
**(B)**			
**True\Pred**	**C**	**PS**	**AS**
C	29	1	0
PS	3	15	2
AS	0	1	19
**(C)**			
**Class**	**Precision**	**Recall**	**F1-Score**
C	0.91	0.97	0.94
PS	0.88	0.75	0.81
AS	0.90	0.95	0.93

Note. 5-fold stratified CV on standardized ln-hormones; exploratory only.

**Table 6:** Birth-weight models (OLS coefficients; PLS CV R^2^). OLS: birth weight (kg) ~ weeks + BMI + sex + ln(hormones) (HC3); Adj. R^2^ = 0.291. PLS (two comps, 5-fold CV): R^2^ = −0.777 → poor predictive value.

Term	β (kg)	SE	*p*
Weeks	0.062	0.026	0.0168
BMI	0.014	0.023	0.538
Sex (Male = 1)	−0.122	0.095	0.201
ln(Estradiol)	0.373	0.238	0.117
ln(Estriol)	0.377	0.482	0.435
ln(Estrone)	−0.125	0.272	0.645
ln(Progesterone)	0.021	0.372	0.955
ln(Testosterone)	−0.146	0.361	0.685
ln(Pregnanediol)	0.315	0.328	0.337

## Data Availability

De-identified data, analysis code, and figure scripts are available from the corresponding author upon reasonable request. The Supplementary Materials (Figures S1 and S2; Tables S1 and S2) provide additional statistics and outputs referenced in the text.

## Supplementary Materials

The supporting information can be downloaded at: https://www.mdpi.com/article/10.3390/ijms262110548/s1.

## Abbreviations

The following abbreviations are used in this manuscript:

DOHaD	Developmental Origins of Health and Disease
BH	Benjamini–Hochberg
AUC	Area Under Curve
OvR	One-versus-rest
OLS	Ordinary Least Squares
SE	Standard Error
PLS	Partial Least Squares
CI	Confidence Interval
ETS	Environmental Tobacco Smoke
CAD	Charged Aerosol Detector
CS	Cigarette Smoke
SIDS	Sudden Infant death syndrome
BMI	body mass index
C	non-smoking control
PS	passive smokers
AS	active smokers
SPE	solid phase extraction
HPLC	high-performance liquid chromatography
SD	standard deviation
LOD	limit of detection
LOQ	limit of quantification
CV	Cross-validation
E1	estrone
E2	estradiol
E3	estriol
T	testosterone
PD	pregnanediol
P	progesterone

## Appendix A

**Table A1:** HPLC dual-mode gradient program.

Time (Min)	Flow Rate (mL/Min)	Acetonitrile (%)
0	1	10
15	1	25
45	1	33
65	1	33
85	1	40
95	1	68
140	1	99

**Table A2:** Accuracy, Precision, and Recovery of the method.

Steroid	Nominal Concentration (ng/mL)	Within Day (n = 5)		Between Day (n = 15)		Recoveries %
		Accuracy %	Precision %	Accuracy %	Precision %	
Estradiol	33 ng	108	12	105	13	83
	50 ng	104	10	101	14	84
	250 ng	102	7	98	9	86
	500 ng	99	4.6	96	7	87
Estriol	30 ng	89	13.5	83	11	82
	50 ng	88	11	86	13	83
	250 ng	92	4.3	90	7	86
	500 ng	96	5	93	6	88
Estrone	33 ng	101	13	91	10	88
	50 ng	100	10	92	8	86
	250 ng	98	8	94	11	89
	500 ng	99	6	98	6	90
Progesterone	30 ng	101	11	98	10	81
	50 ng	98	12	97	8	80
	250 ng	99	10	92	6	83
	500 ng	100	9	99	4	85
Testosterone	30 ng	103	12	98	13	90
	50 ng	101	8	94	9	91
	250 ng	98	7	93	10	93
	500 ng	97	4	95	6	94
Pregnanediol	30 ng	87	11	83	9	78
	50 ng	88	10	85	7	80
	250 ng	92	8	90	4	82
	500 ng	90	5	91	5	86
