# Supplementary material for: Targeted Analysis of Placental Steroid Hormones in Relation to Maternal Tobacco Smoke Exposure: Early Markers Relevant to DOHaD (Developmental Origins of Health and Disease)
Source: Int J Mol Sci. 2025 Oct 30;26(21):10548. doi: 10.3390/ijms262110548 (PMC12609261; doi:10.3390/ijms262110548)
Supplement: Supplementary file 1 [file ijms-26-10548-s001.zip › ijms-3895085-supplementary/Supplementary_Table_S1.docx]

**Table S1.** Model B (Model A + birth weight): % change [95% CI] vs controls with HC3--robust p--values.

**Model B** (Model A + birth weight)

| **Hormone** | **AS vs C** | **PS vs C** |
| --- | --- | --- |
| Estradiol | −46.5% [−53.4; −38.5] (*p* = 1.12 × 10^−^18) | −31.4% [−38.4; −23.5] (*p* = 7.91 × 10^−^12) |
| Estriol | −22.9% [−30.1; −14.9] (*p* = 2.64 × 10^−^7) | −10.4% [−16.9; −3.3] (*p* = 0.00471) |
| Estrone | −29.5% [−36.9; −21.4] (p = 4.01 × 10^−^10) | −21.0% [−30.1; −10.8] (*p* = 0.000155) |
| Progesterone | −27.0% [−41.3; −9.2] (*p* = 0.00462) | −10.4% [−18.5; −1.4] (*p* = 0.0241) |
| Testosterone | +40.1% [ + 20.2; +63.3] (*p* = 1.56 × 10^−^5) | +20.8% [+8.9; +33.9] (*p* = 0.000343) |
| Pregnanediol | −31.4% [−37.2; −25.1] (*p* = 4.94 × 10^−^17) | −9.3% [−14.5; −3.8] (*p* = 0.00119) |
