# Supplementary material for: Targeted Analysis of Placental Steroid Hormones in Relation to Maternal Tobacco Smoke Exposure: Early Markers Relevant to DOHaD (Developmental Origins of Health and Disease)
Source: Int J Mol Sci. 2025 Oct 30;26(21):10548. doi: 10.3390/ijms262110548 (PMC12609261; doi:10.3390/ijms262110548)
Supplement: Supplementary file 1 [file ijms-26-10548-s001.zip › ijms-3895085-supplementary/Supplementary_Table_S2_Regression_Coefficients.docx]

# Supplementary Table S2. Regression coefficients for Models A and B

Model specification: Model A adjusts for gestational weeks, pre-pregnancy maternal BMI, and newborn sex. Model B additionally adjusts for birth weight (kg). Coefficients (β) and standard errors (SE) are on the ln-scale; percent changes are back-transformed as (exp(β) − 1) × 100 with 95% CIs derived from β ± 1.96·SE.

The tables below report the group contrasts (AS vs C and PS vs C). Covariates are included as adjustment terms; full model code and data are available in the public repository (DOI to be provided at acceptance).

## Model A — Group contrasts (HC3)

| Hormone | Contrast (vs C) | beta (ln scale) | SE (HC3) | p-value | % change [95% CI] |
| --- | --- | --- | --- | --- | --- |
| Estradiol | AS | -0.6205 | 0.0649 | 1.23e-21 | -46.2% [-52.7; -38.9] |
| Estradiol | PS | -0.3723 | 0.0440 | 2.78e-17 | -31.1% [-36.8; -24.9] |
| Estriol | AS | -0.2839 | 0.0425 | 2.35e-11 | -24.7% [-30.7; -18.2] |
| Estriol | PS | -0.1313 | 0.0320 | 4.02e-05 | -12.3% [-17.6; -6.6] |
| Estrone | AS | -0.3003 | 0.0471 | 1.78e-10 | -25.9% [-32.5; -18.8] |
| Estrone | PS | -0.1913 | 0.0493 | 0.000103 | -17.4% [-25.0; -9.0] |
| Progesterone | AS | -0.3309 | 0.1161 | 0.00436 | -28.2% [-42.8; -9.8] |
| Progesterone | PS | -0.1242 | 0.0297 | 2.84e-05 | -11.7% [-16.7; -6.4] |
| Testosterone | AS | 0.3421 | 0.0544 | 3.27e-10 | 40.8% [26.5; 56.6] |
| Testosterone | PS | 0.1928 | 0.0476 | 5.21e-05 | 21.3% [10.4; 33.1] |
| Pregnanediol | AS | -0.3772 | 0.0409 | 2.76e-20 | -31.4% [-36.7; -25.7] |
| Pregnanediol | PS | -0.0973 | 0.0248 | 8.78e-05 | -9.3% [-13.6; -4.8] |

## Model B — Group contrasts (HC3)

| Hormone | Contrast (vs C) | beta (ln scale) | SE (HC3) | p-value | % change [95% CI] |
| --- | --- | --- | --- | --- | --- |
| Estradiol | AS | -0.6251 | 0.0709 | 1.12e-18 | -46.5% [-53.4; -38.5] |
| Estradiol | PS | -0.3764 | 0.0550 | 7.91e-12 | -31.4% [-38.4; -23.5] |
| Estriol | AS | -0.2597 | 0.0505 | 2.64e-07 | -22.9% [-30.1; -14.9] |
| Estriol | PS | -0.1094 | 0.0387 | 0.00471 | -10.4% [-16.9; -3.3] |
| Estrone | AS | -0.3502 | 0.0560 | 4.01e-10 | -29.5% [-36.9; -21.4] |
| Estrone | PS | -0.2363 | 0.0625 | 0.000155 | -21.0% [-30.1; -10.8] |
| Progesterone | AS | -0.3144 | 0.1110 | 0.00462 | -27.0% [-41.3; -9.2] |
| Progesterone | PS | -0.1093 | 0.0484 | 0.0241 | -10.4% [-18.5; -1.4] |
| Testosterone | AS | 0.3375 | 0.0781 | 1.56e-05 | 40.1% [20.2; 63.3] |
| Testosterone | PS | 0.1886 | 0.0527 | 0.000343 | 20.8% [8.9; 33.9] |
| Pregnanediol | AS | -0.3772 | 0.0450 | 4.94e-17 | -31.4% [-37.2; -25.1] |
| Pregnanediol | PS | -0.0973 | 0.0300 | 0.00119 | -9.3% [-14.5; -3.8] |

Abbreviations: C = controls (non-smokers); PS = passive smokers; AS = active smokers; HC3 = heteroskedasticity-consistent standard errors (type 3).
