# Supplementary figures and images for: Targeted Analysis of Placental Steroid Hormones in Relation to Maternal Tobacco Smoke Exposure: Early Markers Relevant to DOHaD (Developmental Origins of Health and Disease)
Source: Int J Mol Sci. 2025 Oct 30;26(21):10548. doi: 10.3390/ijms262110548 (PMC12609261; doi:10.3390/ijms262110548)

# Adjusted effects (AS vs C) — Model B (supplement)

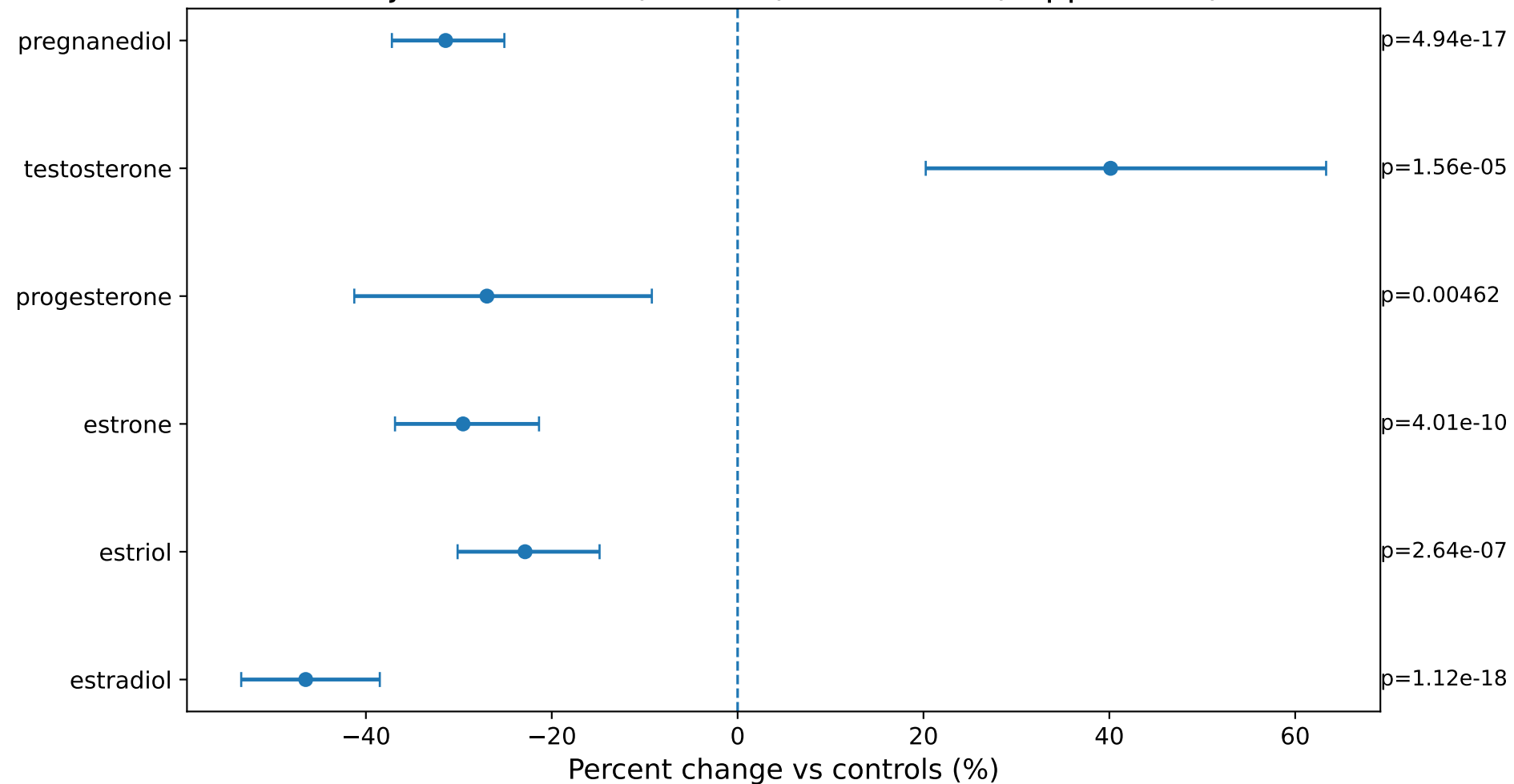

Supplement: Supplementary file 1 [file ijms-26-10548-s001.zip › ijms-3895085-supplementary/Supp_Figure_S1_Forest_AS_ModelB.pdf]

# Adjusted effects (PS vs C) — Model B (supplement)

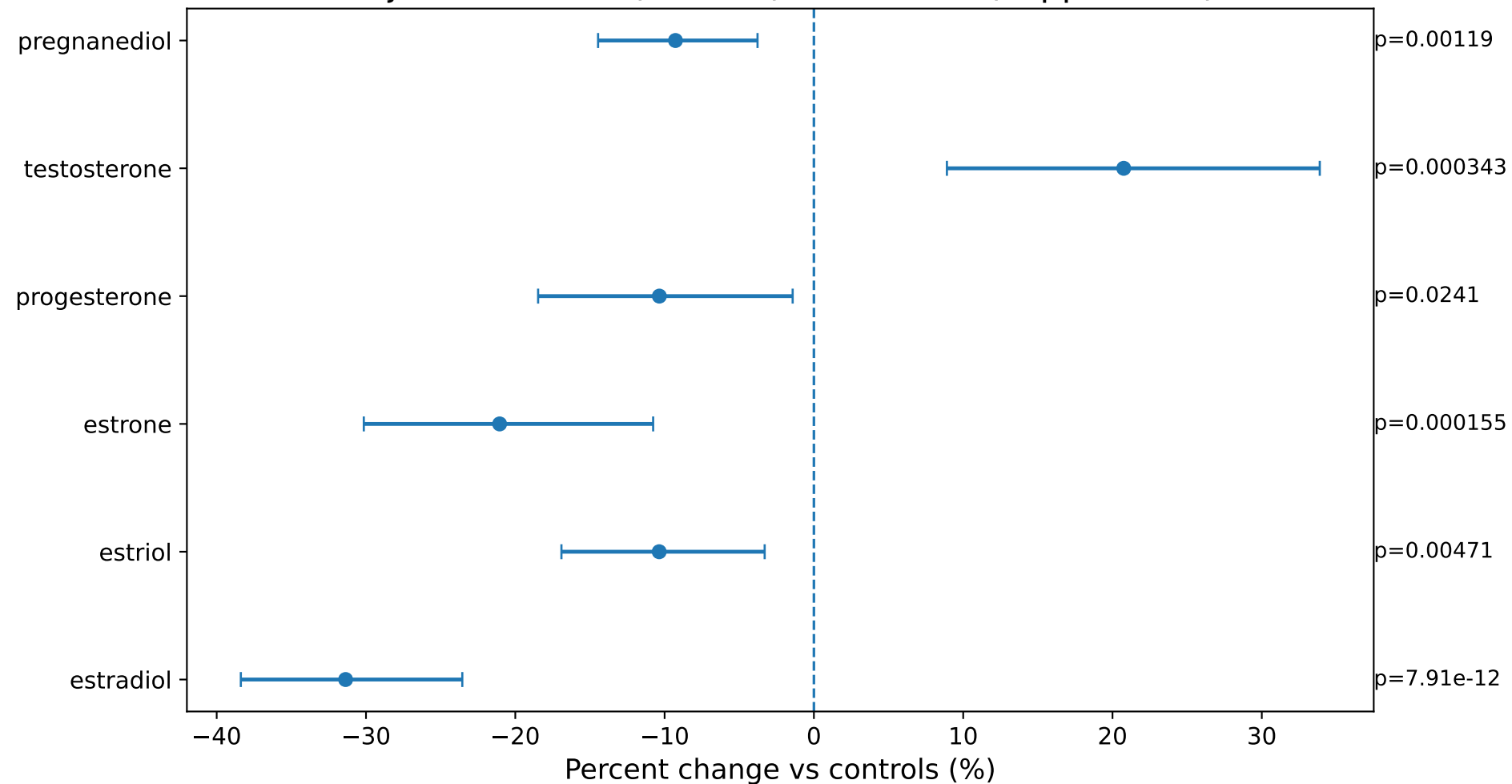

Supplement: Supplementary file 1 [file ijms-26-10548-s001.zip › ijms-3895085-supplementary/Supp_Figure_S2_Forest_PS_ModelB.pdf]
